# The Role of Lysophospholipid Metabolites LPC and LPA in the Pathogenesis of Chronic Obstructive Pulmonary Disease

**DOI:** 10.3390/metabo14060317

**Published:** 2024-05-31

**Authors:** Qiqiang Zhou, Yahong Chen, Ying Liang, Yongchang Sun

**Affiliations:** 1 Department of Respiratory and Critical Care Medicine, Peking University Third Hospital, Beijing 100191, China; zhouqqdr@163.com (Q.Z.); chenyahong@vip.sina.com (Y.C.); suny@bjmu.edu.cn (Y.S.); 2 Research Center for Chronic Airway Diseases, Peking University Health Science Center, Beijing 100191, China

**Keywords:** COPD, lipid metabolism, lysophospholipids, lysophosphatidylcholine, lysophosphatidic acid

## Abstract

Chronic obstructive pulmonary disease (COPD) is a heterogeneous lung condition characterized by persistent respiratory symptoms and airflow limitation. While there are some available treatment options, the effectiveness of treatment varies depending on individual differences and the phenotypes of the disease. Therefore, exploring or identifying potential therapeutic targets for COPD is urgently needed. In recent years, there has been growing evidence showing that lysophospholipids, namely lysophosphatidylcholine (LPC) and lysophosphatidic acid (LPA), can play a significant role in the pathogenesis of COPD. Exploring the metabolism of lysophospholipids holds promise for understanding the underlying mechanism of COPD development and developing novel strategies for COPD treatment. This review primarily concentrates on the involvement and signaling pathways of LPC and LPA in the development and progression of COPD. Furthermore, we reviewed their associations with clinical manifestations, phenotypes, and prognosis within the COPD context and discussed the potential of the pivotal signaling molecules as viable therapeutic targets for COPD treatment.

## 1. Introduction

COPD is a heterogeneous lung condition characterized by persistent respiratory symptoms due to abnormalities of the airways and/or alveoli that cause irreversible airflow obstruction [1]. The primary environmental factors contributing to COPD are cigarette smoking and the inhalation of toxic particles and gases, often produced by indoor and outdoor air pollution. With a global prevalence rate now at 10.3%, COPD has become a significant worldwide public health concern. In China, the prevalence of COPD among individuals aged 40 and above was 13.7%, affecting approximately 100 million people [2]. Annually, COPD claims the lives of over 3 million individuals worldwide [3], making it one of the top three leading causes of death globally, with 90% of these deaths occurring in low- and middle-income countries [1]. COPD has a substantial impact on national health systems, imposing a considerable economic and health burden on individuals, families, and communities [4]. This burden is expected to escalate in the coming decades due to continued exposure to COPD risk factors and an aging population [1]. Various key factors are involved in the pathogenesis and progression of COPD, including chronic airway inflammation and emphysema, oxidative stress, apoptosis, imbalances between proteases and antiproteases, as well as airway remodeling and fibrosis [5,6].

Lipids serve multiple functions within cells, acting as major cellular components and important energy storage reservoirs. Moreover, extensive research conducted over the past two decades has elucidated their role in cellular signaling [7]. Recent studies have underscored the significance of lipid metabolism in COPD pathogenesis [8], with particular attention given to lysophospholipids. Lysophospholipids are essential lipid components, representing monoacyl hydrolyzed products derived from diacyl phospholipid precursors, aptly named for their erythrocyte-lysing capability [4]. These lysophospholipids participate in numerous signaling pathways and play an important and complicated role in the pathogenesis of COPD. Previous research has predominantly centered around two lysophospholipids: lysophosphatidic acid (LPA) and lysophosphatidylcholine (LPC), the most abundant lysophospholipid in the human body. In this review, we provide an overall picture of the roles of the LPC–LPA axis in the pathogenesis of COPD. This study aims to deepen our understanding of COPD pathogenesis and open up new avenues for future therapeutic strategies.

## 2. Lysophospholipids

Lysophospholipids represent a common class of phospholipids, encompassing LPC, lysophosphatidylethanolamine (LPE), lysophosphatidylserine (LPS), lysophosphatidylinositol (LPI), lysophosphatidylglycerol (LPG), and LPA. These lysophospholipids are produced through the hydrolysis of membrane phospholipids by either phospholipase A1 or phospholipase A2 enzymes [9]. In circulation, LPC is the most prevalent lysophospholipid [10], followed by LPE and LPA. In the physiological environment, lysophospholipids are abundantly present in the extracellular space, such as plasma and interstitial fluid. Their amphiphilic nature allows certain molecules to be secreted extracellularly, where they serve as signaling molecules [9]. Among the lysophospholipids, LPA and LPC are the most extensively studied. LPA is composed of a phosphate group, a central glycerol backbone, and a fatty acid chain. LPA predominantly originates extracellularly, primarily through various pathways. Notably, phospholipids within biological membranes, such as phosphatidylcholine (PC), phosphatidylethanolamine (PE), or phosphatidylserine (PS), can be enzymatically hydrolyzed by phospholipase D (PLD) 1 or 2 to generate phosphatidic acid (PA). In an alternative pathway, intracellular diacylglycerol (DAG) undergoes conversion to PA catalyzed by DAG kinase (DAGK). Subsequently, PA is transformed into LPA through the actions of PA-selective phospholipase (PL) A1 or PLA2 enzymes localized at the cell membrane surface [11]. Enzymes responsible for PA production, such as phospholipase D and diacylglycerol kinase, are exclusively found within the cytoplasm. Consequently, the production of LPA via PA-selective PLA is believed to be closely linked to PA production and its subsequent transport [12]. Moreover, intracellularly, LPA can be produced through acylglycerol kinase (AGK) acting on monoacylglycerol, and via acylation of glycerol-3-phosphate by glycerol-3-phosphate acyltransferase. This intracellular pathway represents a significant mechanism for LPA production [13,14].

**Figure 1:**
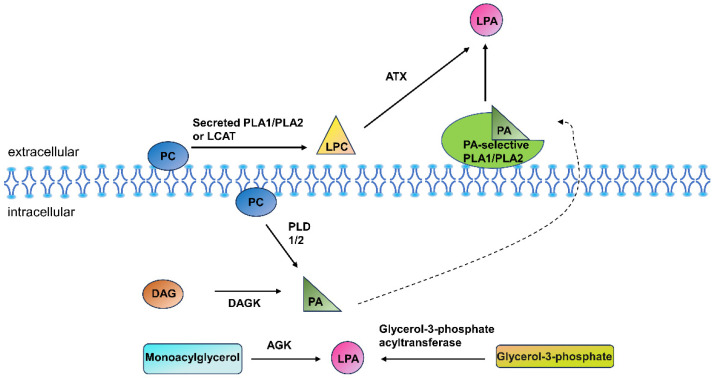
Lysophosphatidic acid (LPA) generation Phospholipids within biological membranes, such as phosphatidylcholine (PC), can be enzymatically hydrolyzed by phospholipase D (PLD) 1 or 2 to generate phosphatidic acid (PA). In an alternative pathway, intracellular diacylglycerol (DAG) undergoes conversion to PA catalyzed by DAG kinase (DAGK). Subsequently, PA produced through both of these pathways is transformed into LPA by PA-specific phospholipase (PL) A1 or PLA2 enzymes localized at the cell membrane surface. Moreover, intracellularly, LPA can be produced through acylglycerol kinase (AGK) acting on monoacylglycerol, and via acylation of glycerol-3-phosphate by glycerol-3-phosphate acyltransferase. Extracellularly, Phospholipids such as PC are converted to lysophospholipids such as lysophosphatidylcholine (LPC) by secretory PLA1 or PLA2. ATX then cleaves the choline of LPC to generate LPA. Additionally, LPA can be generated from phospholipids through a sequential process involving lecithin cholesterol acyltransferase (LCAT), followed by the action of autotaxin (ATX).

Extracellularly, phospholipids, such as PC, are converted into lysophospholipids, such as LPC. This conversion is facilitated by secretory PLA2 or phospholipase PLA1 enzymes. Autotaxin (ATX), an abundantly secreted lysophospholipase D found extracellularly, plays a pivotal role in the following process. ATX cleaves the choline, ethanolamine, or serine portions of LPC, LPE, and LPS, respectively, to generate LPA [13]. ATX, present in plasma, is responsible for regulating plasma LPA levels [15]. Additionally, LPA can be generated from phospholipids through a sequential action involving lecithin cholesterol acyltransferase (LCAT) followed by ATX [16,17] (Figure 1).

Extracellular LPA circulates and can bind to one of six subtypes of LPA receptors (LPARs) denoted as LPAR1-6, all of which belong to the G protein-coupled receptor (GPCR) family [18]. These LPARs can activate downstream signaling effectors, including Rho-associated kinase (Rock), phospholipase C (PLC), inositol triphosphate (IP3), diacylglycerol (DAG), mitogen-activated protein kinase (MAPK), Phosphoinositide-3-kinase (PI3K), protein kinase b (AKT), and adenylyl cyclase (AC)/cAMP, through various G-protein subunits, such as Gα12/13, Gαq/11, Gαi/o, and Gαs [19,20,21]. The activation of Gα12/13 promotes the Rho/Rock and Rho/ Serum Response Factor (SRF) pathways, responsible for orchestrating cell motility, infiltration, and cytoskeletal rearrangements. Gαq/11 activation stimulates the IP3 pathway, facilitating vasodilation and governing cell growth and immune responses. Gαs activation mediates AC signaling, culminating in cyclic AMP (cAMP) production. Meanwhile, Gαi/o activation supports various cellular processes including morphological alterations, cell migration, and survival through PLC, Ras/MAPK, PI3K/Rac, and PI3K/Akt pathways [22] (Table 1). LPA initiates a diverse range of cellular processes, encompassing cell proliferation, apoptosis inhibition, cell migration (including T-cells, neural cells, fibroblasts, and tumor cells), secretion of cytokines (including Interleukin (IL)-2, IL-8, IL-12, and Tumor necrosis factor α), platelet aggregation, smooth muscle contraction, and myofibroblast differentiation [17,22,23,24,25,26]. These biological effects underscore the significant role of LPA as a crucial molecule in cellular signaling and functional regulation.

## 3. LPA, LPC Receptors in the Respiratory System

### 3.1. LPA

LPAR1-3 have broad expression across lung epithelial cells, endothelial cells, airway smooth muscle cells, and various immune cell types, including eosinophils, macrophages, neutrophils, and lymphocytes [26]. Particularly in human airway epithelial cells and airway smooth muscle cells, studies have confirmed the presence of LPAR1, LPAR2, and LPAR3 receptors [23,27]. Flow cytometry-based investigations have also revealed the surface expression of LPAR1, LPAR2, and LPAR3 receptors on both Type 1 T helper (Th1) and Type 2 T helper (Th2) cells [13,27,28]. Conversely, eosinophils express LPAR1 and LPAR3 but not LPAR2 [13,29].

LPA exerts a range of effects on airway cells, including enhancing smooth muscle cell contractility, promoting the proliferation of mesenchymal cells, reinforcing the epithelial cell barrier, stimulating the expression of both pro- and anti-inflammatory cytokines in human bronchial epithelial cells, and facilitating T cell homing. These findings underscore the distinct biological roles that LPA plays across various lung cell types [13,25,30].

### 3.2. LPC

Compared to LPA, the receptors of LPC are not well studied. The main LPC receptors that have been reported are GPCR and Toll-like receptors (TLR). In GRCP, GPR132 (G2A) and GPR4 are known LPC receptors. The affinity of LPC for G2A is significantly higher than that for GPR4. G2A is mainly expressed in lymphocytes and macrophages [31].

When LPC binds to G2A, it activates extracellular signal-regulated kinase (ERK) mitogen-activated protein kinase to induce T lymphocyte and macrophage migration [32,33]. In addition, LPC can activate nuclear factor kappa B (NF-kB), p38 MAPK, and c-Jun N-terminal kinase (JUK) signaling pathways by binding to TLR2 and TLR4 receptors [34,35]. The activation of these pathways induces the production of pro-inflammatory factors that modulate inflammation. These activations mediate a wide array of biological functions, such as the induction of chemotaxis, modulation of inflammatory factors, regulation of oxidative stress, and apoptosis [36]. It is worth noting, however, that the specific receptors for LPC and their expression in the lungs remain poorly characterized. Therefore, future studies should prioritize these issues in order to gain a more thorough understanding of LPC’s biological role and its potential implications in chronic airway diseases.

## 4. Role of LPC–LPA Axis in COPD Pathogenesis

### 4.1. Chronic Airway Inflammation 

It is well-established that chronic airway inflammation in COPD primarily involves inflammatory cells such as airway neutrophils, macrophages, T-lymphocytes, and various inflammatory cytokines [37]. LPC and LPA actively participate in the development and maintenance of chronic airway inflammation in COPD by modulating the activities of the aforementioned inflammatory cells and cytokines.

LPA appears to have dual roles, with both pro-inflammatory and anti-inflammatory effects in chronic airway inflammation. LPA has been found to strongly stimulate IL-8 secretion in various airway epithelial cell types, consequently increasing airway neutrophil infiltration [25,38]. The LPA-induced IL-8 secretion process is mediated through LPAR 1-3, coupled to Gαi and Gα12/13, in human bronchial epithelial cells [38,39,40]. This regulation involves changes in intracellular calcium ion concentration ([Ca^2+^]i), IκB phosphorylation, NF-κB activation, and the transcriptional activation of the IL-8 gene in human bronchial epithelial cells (HBEpCs) [41]. In line with in vitro findings, the intratracheal injection of LPA in mice led to increased levels of MIP-2 (the murine homolog of IL-8) within 3 h and chemoattracted neutrophil infiltration in the alveolar lumen within 6 h [25]. Furthermore, LPA significantly enhanced the chemotaxis of neutrophils isolated from patients with pneumonia [42,43]. These results collectively indicate that LPA plays a role in the regulation of airway inflammation by stimulating the release of chemokines and other inflammatory mediators, as well as the infiltration of neutrophils in the airway [25]. 

**Figure 2:**
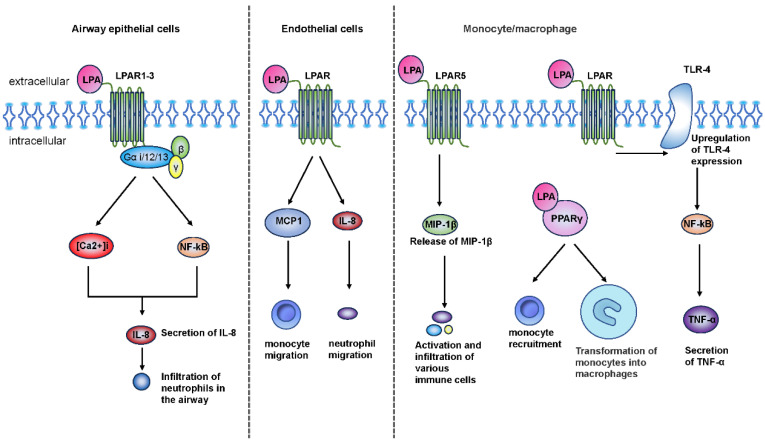
The role of lysophosphatidic acid (LPA) in airway inflammation. LPA can strongly stimulate the secretion of IL-8 in the various airway epithelial cells and increase the infiltration of airway neutrophils. LPA-induced IL-8 secretion is mediated through Gαi and Gα12/13-coupled LPAR1-3 in human bronchial epithelial cells, partially mediated by [Ca^2+^]i, IκB phosphorylation, and NF-κB activation Changes and regulation of transcriptional activation of IL-8 gene expression in human bronchial epithelial cells (HBEpC). In addition, LPA promotes the recruitment of monocytes and mediates the differentiation of monocytes into macrophages. This process is likely through the activation of peroxisome proliferators-activated receptor γ (PPARγ), a non-canonical LPA receptor. LPA also indirectly regulates monocyte and neutrophil migration through the production of chemokines monocyte chemotactic protein 1 (MCP1) and IL-8 by endothelial cells. In addition, LPA acts on LPAR5 to induce the release of macrophage inflammatory protein 1 β (MIP-1β), a potent chemoattractant and activator of monocytes, lymphocytes, and various immune cells. Simultaneously, LPA significantly upregulated Toll-like receptors 4 (TLR-4) expression and promoted NF-kB activation, thereby prompting THP-1 cells (a human monocytic cell line) to secrete the pro-inflammatory cytokine TNF-α.

Furthermore, LPA plays a role in promoting monocyte recruitment and mediating monocyte differentiation into macrophages in both humans and mice [44,45,46]. This process may be mediated through the activation of peroxisome proliferators-activated receptor γ (PPARγ), which acts as a non-classical LPAR [45]. LPA also indirectly regulates monocytes and neutrophil migration via stimulating the production of the chemokines monocyte chemotactic protein 1 (MCP1) and IL-8 by endothelial cells [47]. Additionally, LPA interacts with LPAR5 to induce the release of macrophage inflammatory protein 1 β (MIP-1β), a potent chemotactic and activating agent for monocytes, lymphocytes, and various immune cells [48]. Simultaneously, LPA significantly upregulates the expression of TLR-4 and promotes NF-kB activation, consequently contributing to the secretion of pro-inflammatory cytokines TNF-α by Tohoku hospital pediatrics-1(THP-1) cells, a human monocyte cell line [49] (Figure 2).

These findings suggest that LPA exhibits a pro-inflammatory activity in airway inflammation of COPD in multiple ways, including promoting neutrophil recruitment, facilitating macrophage migration, and inducing cytokine secretion.

**Figure 3:**
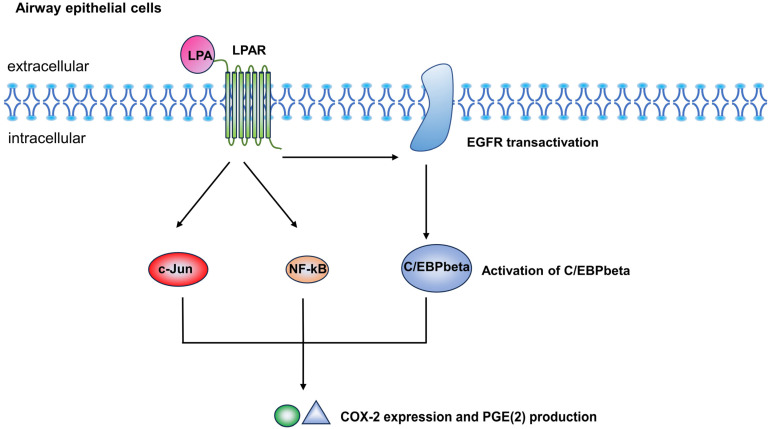
The anti-inflammatory role of lysophosphatidic acid (LPA) in airway inflammation. LPA induces COX-2 expression and PGE(2) production through EGFR transactivation-independent activation of transcriptional factors NF-kappaB and c-Jun, and EGFR transactivation-dependent activation of C/EBPbeta in HBEpCs.

However, it is important to note that LPA might also exhibit an anti-inflammatory role in airway inflammation. LPA induces the expression of Cyclooxygenase (COX)-2 and the production of Prostaglandin E2 (PGE2) through regulating transcription factors such as NF-κB, c-Jun, and the epidermal growth factor receptor (EGFR) trans-activation-dependent activation of CCAAT/enhancer-binding protein β (C/EBPβ) in HBEpCs [50]. In the lungs, unlike many other parts of the body, PGE2 and COX2 are involved in limiting the immune inflammatory response as well as contributing to tissue repair processes [51,52]. Thus, the LPA-induced enhancement of COX-2 expression and PGE2 release may have a protective role against airway inflammation [38] (Figure 3).

Therefore, the role of LPA in airway inflammation is complex, as it can trigger an inflammatory response through the infiltration of neutrophils, the migration of monocytes, macrophages, and lymphocytes, and the secretion of pro-inflammatory cytokines, while also exerting anti-inflammatory effects by inducing COX-2 expression and promoting PGE2 release.

LPC is the precursor of LPA and can increase the expression of chemokines such as MCP-1 and IL-8 in endothelial cells, thereby promoting the migration of monocytes, macrophages, neutrophils, and lymphocytes [4,53]. Moreover, LPC significantly enhances the expression of chemokine receptors CXC chemokine receptor 4 (CXCR4) and cysteine-cysteine chemokine receptor 5 (CCR5) in human CD4 T-cell lines and induces the expression of CXCR4 and C-X-C motif chemokine ligand 12(CXCL12) in monocytes, thereby augmenting their migratory capacity [54,55]. The mechanism by which LPC induces monocyte migration primarily involves the activation of the protein kinase D 2 (PKD2)/P38MAPK signaling pathway and Ca^2+^ ion channels [31,56,57]. Additionally, LPC increases the release of inflammatory factors, including IL-1β, IL-8, Interferons-γ(IFNγ), IL-6, IL-5, and arachidonic acid (AA) [58,59].

However, different LPC subtypes may have varying effects, which can be influenced by the length and saturation of the fatty acid chain. Saturated LPCs, such as LPC (16:0), induce an inflammatory response, contributing to immune cell migration and the release of pro-inflammatory factors. In contrast, polyunsaturated LPCs, such as LPC (20:4), LPC (20:5), and LPC (22:6), can serve as potent anti-inflammatory agents counteracting the immune responses triggered by saturated LPCs [31]. LPCs can downregulate the production of pro-inflammatory mediators (e.g., IL-5, IL-6, NO, TNF-α) and upregulate the expression of anti-inflammatory mediators (e.g., IL-4 and IL-10) [31,60], resulting in an anti-inflammatory effect. These results showed that different LPC subtypes play distinct roles in the regulation of inflammation, with some subtypes promoting inflammatory processes while others exhibit anti-inflammatory effects [61]. The underlying mechanism remains unclear and needs further research to address this issue. Currently, there is a notable paucity of research on the associations between LPC, LPA, and inflammation, specifically regarding cytokine profiles within COPD cohorts. This area warrants further investigation in future studies.

### 4.2. Airway Remodeling and Fibrosis

There is growing evidence to support the association of airway remodeling, fibrosis, and epithelial-mesenchymal transition (EMT) with airway constriction and the progression of emphysema [62,63]. LPA can trigger an inflammatory growth factor-like response and promote cell proliferation [64]. Specifically, LPA can independently stimulate the proliferation of human airway smooth muscle (HASM) cells or synergize with epidermal growth factor (EGF) to further enhance HASM cell proliferation [65,66]. Additionally, LPA induces actin reorganization through Gαi-2 and Gαq proteins, leading to cAMP accumulation, PI hydrolysis, and Rho activation in HASM cells. This, in turn, results in HASM cell proliferation in a concentration-dependent manner [67].

Furthermore, LPA stimulates migration, fibronectin secretion, and filamentous pseudopod extension in human bronchial epithelial cells through the activation of protein kinase Cδ (PKCδ) and cortactin phosphorylation [23,68,69]. LPA also mediates fibroblast migration and proliferation via LPAR1 and is implicated in collagen gel contraction, a process associated with the development of pulmonary fibrosis [68]. Elevated LPA levels in the airways have been observed in a mouse bleomycin-induced idiopathic pulmonary fibrosis (IPF) model [70]. LPA levels in the airways were elevated in a mouse bleomycin IPF model [71]. The inhibition of LPA receptor 1 and antagonism of ATX reduced fibroblast chemotaxis in response to bronchoalveolar lavage (BAL) fluid, ultimately preventing pulmonary fibrosis [71,72]. Regarding LPC, there is currently no direct evidence linking it to airway remodeling in patients or animal models with COPD.

### 4.3. Apoptosis

Elevated levels of apoptotic cells in the airways of COPD patients represent one of the notable pathological features of the disease [73]. Apoptosis has been proven to be involved in the development of emphysema [74].

It is widely recognized that LPA has been identified as a promoter of proliferation in various airway cell types, including HASM cells and lung fibroblasts [75]. However, the role of LPA in cell survival and apoptosis is exceedingly intricate and hinges on numerous factors, including concentration, receptor type, cell type, and physiological context. Research has demonstrated that low concentrations of LPA stimulate the proliferation of HASM cells, whereas high concentrations (greater than 100 μmol/mL) of LPA induce apoptosis [76]. Hence, the role of LPA may exhibit distinct effects in different scenarios. This underscores the necessity to consider these factors when investigating the apoptosis mechanism of LPA in COPD. In the pathological context of COPD, whether the concentration of LPA is elevated and potentially triggers apoptosis in alveolar cells warrants further investigation. Additionally, studies have revealed that LPA signaling promotes apoptosis in lung epithelial cells following bleomycin injury through its receptor LPAR1. Notably, after the bleomycin challenge, LPAR1, and LPAR2-deficient mice exhibited significantly reduced numbers of apoptotic cells in the alveolar and bronchial epithelium, along with decreased lung caspase-3 activity [71,77]. In line with these in vivo findings, LPA signaling through LPAR1 was found to induce apoptosis in cultured normal human bronchial epithelial cells [78].

Moreover, the increased presence of apoptotic cells in the airways of COPD patients may stem from a deficiency in the ability of alveolar macrophages to phagocytize apoptotic cells, a process known as efferocytosis, particularly in COPD patients and current smokers [79]. This defect in efferocytosis is considered one of the principal mechanisms driving the progression of emphysema. Disruptions in exocytosis may lead to heightened inflammation, triggering the release of proteases and resulting in excessive apoptosis and the necrosis of lung cells [80], ultimately contributing to emphysema development [81]. Indeed, there is evidence demonstrating that LPA induces impaired exocytosis in airway macrophages of COPD patients by activating RhoA signaling [82].

Regarding whether LPC can regulate lung tissue apoptosis in COPD, current research has not provided a direct answer. However, LPC has been demonstrated to induce apoptosis in various cell types, including endothelial cells, cardiomyocytes [31,83], coronary smooth muscle cells [84], and ovarian cells [85]. The mechanisms underlying LPC-induced apoptosis involve caspase activation, calcium influx, cytochrome c release, and the mitochondrial pathway [31]. Additionally, LPC can induce apoptosis by upregulating the expression of the Fas ligand (FasL) through the activation of the NF-κB signaling pathway [59].

In an emphysema model, lysophosphatidylcholine acyltransferase 1 (LPCAT1), an enzyme responsible for catalyzing surfactant lipid biosynthesis and expressed in type 2 alveolar epithelial cells, converts LPC to PC, thereby reducing LPC levels in the body. It was observed that Lpcat1 knockout (KO) mice were more susceptible to porcine pancreatic elastase (PPE)-induced emphysema. Significantly, the addition of artificial surfactants did not reverse the aggravation of emphysema, ruling out surfactant damage as a contributing factor to emphysema. Simultaneously, LPCAT1 KO attenuated the proliferation of lung epithelial cells and enhanced apoptosis induced by cigarette smoke extract (CSE). Therefore, the protective effect of LPCAT1 on emphysema may be linked to the inhibition of apoptosis in alveolar type 2 (AEC2) cells [86].

Considering that LPCAT1 is the enzyme responsible for the conversion of LPC, some studies have also suggested that LPC can induce various forms of cell damage and apoptosis. Thus, it is speculated that the promotion of alveolar cell apoptosis by LPCAT1 KO might result from excessive LPC accumulation. Further research is needed to elucidate the mechanism underlying LPCAT1 deficiency-induced apoptosis. However, some studies have found that LPC can inhibit apoptosis through indirect effects. Apoptotic cells release LPC through the caspase-3-mediated activation of calcium-independent phospholipase A2, and LPC can attract THP1 macrophages to migrate to apoptotic cells, facilitating the clearance of apoptotic cells (efferocytosis), which contributes to the reduction of apoptosis [87].

### 4.4. Oxidative Stress

One of the pathophysiological features of COPD is increased production of reactive oxygen species (ROS) and an imbalance in the redox state of lung tissue. These ROS originate primarily from two sources within cells: mitochondrial oxidative phosphorylation and the activation of nicotinamide adenine dinucleotide phosphate (NADPH) oxidase (NOX) [88].

In vascular endothelial cells, LPC induces injury to human umbilical vein endothelial cells (HUVECs) in a concentration-dependent manner. LPC leads to an overproduction of nitric oxide (NO) and ROS in HUVECs [89]. The use of eNOS inhibitors (L-NAME) and antioxidants significantly inhibits LPC-induced damage to HUVECs [83]. Furthermore, LPC induces the production of ROS through NOX in aortic endothelial cells [90]. LPC also modulates the activity of transcription factors involved in the regulation of oxidative stress gene expression, such as activator protein-1 (AP-1) and NF-kB, which further exacerbates the biological effects of oxidative stress [91]. Additionally, LPC strongly induces the production of NADPH oxidase and superoxide in neutrophils [31]. It is worth noting that unsaturated LPC substances induce persistent superoxide production by neutrophils, while saturated LPC, especially the most abundant 16:0 species, induces significantly lower superoxide production than unsaturated species [92]. These results suggest that LPC may exacerbate oxidative stress by activating neutrophil NADPH oxidase and that there are significant acyl chain-dependent differences in the cellular effects of LPC.

Regarding LPA, a recent study reported that in lung endothelial cells and alveolar macrophages, Prdx6-PLA2 (peroxiredoxin 6 phospholipase A2 activity) stimulates ROS production by converting LPC to LPA, thereby activating the enzyme GTPase, which in turn regulates NOX2 activation [93]. Further experiments showed that ROS production could be reduced by blocking LPA receptors or knocking down LPAR1 [89]. These results emphasize the critical role of the LPC–LPA axis in the regulation of oxidative stress and ROS production, but its specific role in COPD and lung tissues warrants further in-depth investigation.

## 5. Association of Lysophospholipids with Clinical Features, Phenotypes, and Prognosis of COPD

In one study, serum LPA (16:0) and LPA (18:2) levels were higher in COPD smokers than in healthy smokers. They were positively correlated with forced expiratory volume in 1 s (FEV1) in male COPD patients but not in females [94].

However, data from another large study did not show a significant correlation between LPAs and post-bronchodilator FEV1 or between LPAs and post-bronchodilator forced expiratory volume in 1 s/forced vital capacity (FEV1/FVC) ratio [95]. This indicates that the relationship between LPAs and lung function in COPD patients may not be consistent across all studies and may depend on various factors. Interestingly, a post hoc analysis from a clinical trial found that patients with low and intermediate levels of LPA (especially LPA (16:0) and LPA (20:4)) had a higher incidence of exacerbation than those with high LPA levels. Additionally, the first exacerbation occurred earlier in patients with lower LPA levels [46]. This suggests that COPD patients with low LPA levels may be at an increased risk of exacerbation. LPA is implicated in immune modulation and the resolution of inflammation. Consequently, the increased incidences of exacerbations and their earlier onset in patients with reduced LPA levels could be related to disruptions of airway immune function or the resolution of inflammation [46]. In the future, it is essential to investigate the specific molecular signaling pathways triggered by different LPA species, as they exhibit different biological activities, especially in airway inflammation. 

In a study involving 115 subjects, there was no significant correlation between FEV1/FVC and LPC in BAL from patients with COPD [96]. Another study using LC-MS on plasma from smokers with COPD found that LPC (16:0) and LPC (18:1) were significantly negatively correlated with the percentage predicted of FEV1 (FEV1%pred) and the ratio of FEV1/FVC [97]. This suggested that increased levels of LPC may be associated with a decline in lung function in COPD, possibly due to its involvement in oxidative stress. Our previous study revealed that LPC (18:3) was significantly lower during acute exacerbations than in the recovery stage in COPD patients, especially in non-eosinophilic exacerbators [4]. This observation might be explained by the anti-inflammatory properties associated with unsaturated LPCs. Additionally, our recent study indicated that LPC (16:0) and LPC (20:2) levels were increased in eosinophilic AECOPD and were associated with certain positive clinical outcomes, including less hypercapnic respiratory failure, shorter intensive care unit (ICU) stays, and lower fibrinogen levels [98], indicating that LPC (16:0) and LPC (20:2) may be linked to the eosinophilic phenotype of AECOPD and a better prognosis (Table 2). Elevated levels of multiple LPCs were also negatively correlated with plasma levels of fibrinogen, indicating a potentially weaker systemic inflammatory response. Therefore, certain subtypes of LPCs may exert a protective effect in eosinophilic AECOPD. The possible mechanism for the association between LPC and eosinophil phenotype of AECOPD involves PLA2, which is highly expressed in eosinophils and can increase LPC levels by cleaving phosphatidylcholine into LPC [99]. Additionally, LPC has been shown to induce eosinophil adhesion and penetration into the airway wall, although this research is primarily based on allergic diseases such as asthma and allergic rhinitis [100,101]. The biological effects of LPC observed in clinical studies appear to diverge significantly from those observed in animal models or in vitro experiments. We hypothesize that this disparity may be attributable to factors such as the local concentration of LPC, the microenvironment, and the length or degree of unsaturation of the fatty acid chain within LPC molecules. These issues warrant elucidation in future research endeavors. Of note, COPD is characterized by its heterogeneity, with intricate risk factors. It is hypothesized that distinct etiologies or phenotypic manifestations of the disease may exhibit unique lysophospholipid metabolic profiles. However, there is currently a paucity of broader research specifically addressing these associations. Future research is necessary to address these gaps and further our understanding of lysophospholipid metabolism in different COPD phenotypes and COPD patients with different risk factors.

## 6. Potential Therapeutic Targets

Targeting lysophospholipids to modulate efferocytosis appears to be a promising strategy for the treatment of COPD. LPA impairs efferocytosis by inducing RhoA signaling. Statins, which are effective cholesterol-lowering drugs, have the ability to block RhoA. Recent studies have investigated the effects of lovastatin on efferocytosis in primary human macrophages, mouse lungs, and human alveolar macrophages taken from patients with COPD. These studies have shown that lovastatin ameliorates impaired efferocytosis triggered by the LPA-induced RhoA signaling pathway. Lovastatin also enhances the efferocytosis of alveolar macrophages in patients with COPD [82]. Therefore, by targeting RhoA signaling downstream of LPA, lovastatin demonstrates therapeutic potential for COPD with impaired efferocytosis. The chemotactic stimulation of fibroblasts by LPA is inhibited by the LPAR1 antagonist Ki16425, demonstrating that the LPA/LPAR1 signaling pathway plays a pivotal role in chemotaxis. In LPAR1 knockout (KO) mice, bleomycin-induced fibrosis is notably reduced, the hydroxyproline content in the lungs significantly decreases, and the 21-day survival rate markedly improves [71]. These results support the potential of LPAR1 as a therapeutic target. Furthermore, the LPAR1/3 antagonist VPC12249 has been shown to alleviate pulmonary fibrosis in a mouse model of radiation-induced lung fibrosis. Following the observation of increased LPAR1/3 expression in mice exposed to 16 Gray radiation, the administration of VPC12249 effectively inhibited both the decline in survival and the progression of pulmonary fibrosis while also reducing the levels of pro-fibrotic cytokines such as transforming growth factor beta (TGFβ) and connective tissue growth factor (CTGF) [102]. Two LPAR1 inhibitors, BMS-986020 and BMS-986278, are currently under clinical trial as innovative therapeutic agents for idiopathic pulmonary fibrosis (IPF). For patients with COPD combined with pulmonary fibrosis, targeting LPA might be an effective treatment strategy in the future. This link presents a promising area for future research; however, it currently lacks sufficient clinical evidence to justify a detailed discussion in this manuscript.

The lysophospholipid signaling axis plays a crucial role in the pathogenesis and progression of COPD. Currently, there are no reports of lysophospholipid signaling-targeted therapy for COPD treatment. A thorough investigation of the lysophospholipid signaling axis is likely to offer a novel target and direction for COPD treatment. Given the intricate nature of lysophospholipid signaling, its actions may depend on factors such as lysophospholipid concentration, saturation, acyl chain length, the receptor it acts upon, and the specific cell type, underscoring the need for precise cellular and receptor targeting, as well as dosage control in drug development.

It is worth noting that since LPA and LPC signaling are also involved in organismal homeostasis, targeted therapies may lead to abnormal responses in normal tissues. Therefore, when developing drugs, special techniques are necessary to achieve precise drug delivery or accumulation in the lungs, such as inhalation administration. Moreover, COPD exhibits significant heterogeneity, indicating that it is crucial to identify which phenotypes or endotypes are probably most responsive to lysophospholipid signaling pathway-targeted therapeutic approaches. Therefore, numerous issues regarding the lysophospholipid signaling pathway in COPD or other airway diseases need to be addressed in further studies.

## 7. Conclusions

Over the last decade, there has been a growing interest in the role of lysophospholipids. Emerging evidence highlights their significance in COPD pathogenesis, particularly through the LPC–LPA axis.

This axis regulates airway inflammation by regulating the lung infiltration of inflammatory cells such as neutrophils, monocyte macrophages, and lymphocytes and the secretion of various cytokines. Additionally, the LPC–LPA axis can also contribute to airway remodeling and fibrosis via stimulating airway smooth muscle cells, epithelial cell migration, fibroblast migration and proliferation, and epithelial-mesenchymal transition in COPD. Lysophospholipids exhibit a complex role in apoptosis, with LPA showing dual effects depending on various factors such as concentration, receptor type, cell type, and the overall physiological condition of the organism. Furthermore, the relationship between LPC and apoptosis in COPD lung cells remains unclear, although some studies have demonstrated the role of LPC in inducing apoptosis in various cell types.

Concerning clinical characteristics and prognosis, certain lysophospholipid subtypes correlate with lung function and COPD outcomes. For instance, low levels of certain LPAs are associated with poorer prognoses [46], possibly due to impaired immune regulation or compromised inflammation resolution. Moreover, different LPC subtypes exhibit distinct associations with COPD severity, phenotypes, and prognosis, suggesting varied roles in disease progression. This highlights the importance of studying LPC subtypes individually.

As COPD currently lacks highly effective treatments, it has become increasingly important to develop drugs targeting different therapeutic pathways. Key receptors in LPC and LPA signal transduction, along with targeted therapy of signaling molecules, may offer effective strategies for COPD treatment. For instance, lovastatin has shown promise in cell experiments for regulating LPA signaling to enhance the efferocytosis of lung macrophages in COPD, warranting further investigation. However, due to the intricate signaling of lysophospholipids, precise cellular and receptor targeting and dosage control, as well as drug delivery, are crucial for drug development.

In summary, the role of lysophospholipid metabolism in the pathogenesis of COPD has attracted more attention in recent years. Different lysophospholipid molecules play unique roles in airway inflammation, including having pro-inflammatory and anti-inflammatory effects. Elucidating the metabolism of lysophospholipids in diverse cellular populations and advancing therapeutic interventions will translate into clinical applications to cope with the challenges of COPD.

## Figures and Tables

**Table 1:** Summary of LPA receptors, downstream signaling pathways, and biological effects.

Receptor	G-Protein Subunit	Downstream Signaling	Specific Biological Functions
LPAR1-6 (GPCR)	Gα12/13	Rock, Rho/SRF	Cell motility, infiltration, cytoskeletal rearrangements
	Gαq/11	PLC, IP3	Vasodilation, cell growth, immune responses
	Gαs	AC/cAMP	Production of cAMP
	Gαi/o	PLC, Ras/MAPK, PI3K/Rac, PI3K/Akt	Morphological alterations, cell migration, survival

GPCR, G protein-coupled receptor; Rock, Rho-associated kinase; SRF, Serum Response Factor; PLC, Phospholipase C; IP3, Inositol triphosphate; AC, Adenylyl cyclase; cAMP, cyclic AMP; MAPK, mitogen-activated protein kinase; PI3K, Phosphoinositide-3-kinase; AKT, protein kinase b.

**Table 2:** Summary of studies investigating LPC and LPA in COPD patients.

Study	Sample Size	LPC Levels (COPD vs. Control)	LPA Levels (COPD vs. Control)	Correlations with Lung Function	Main Findings
Naz et al. (2017) [94]	Healthy smokers (*n* = 40) and COPD Patients (*n* = 38)	N/A	LPA (16:0) and LPA (18:2) were increased significantly (*p* < 0.05).	LPA (16:0) and LPA (18:2) were correlated with FEV1 in male COPD patients but not in females.	LPA (16:0) and LPA (18:2) were increased significantly in COPD patients and correlated with FEV1 in male COPD patients but not in females.
Li et al. (2021) [95]	Two cohorts of samples, a small cohort, healthy controls (*n* = 10) and COPD patients (*n* = 11). A large cohort, COPD patients (*n* = 268).	N/A	LPA(16:0), LPA(18:0), LPA(18:1), LPA(18:2) were increased significantly (*p* < 0.05).	No correlation with LPA and FEV1 or FEV1/FVC.	The correlation was not significant between LPA and FEV1, or LPA and postbronchodilator ratio FEV1/FVC in either female or male patients.
Li et al. (2021) [46]	COPD patients (*n* = 136)	N/A	N/A	N/A	Patients with low and intermediate levels of LPA (especially LPA (16:0) and LPA (20:4)) had a higher incidence of exacerbation than those with high LPA levels.
Cruickshank-Quinn et al. (2018) [97]	COPD patients (*n* = 149)	N/A	N/A	LPC (16:0) and LPC (18:1) were negatively correlated with the FEV1%pred and the ratio of FEV1/FVC	LPC (16:0) and LPC (18:1) were negatively correlated with the FEV1%pred and the ratio of FEV1/FVC
Eitan Halper-Stromberg et al. (2019) [96]	COPD Patients (*n* = 47)	N/A	N/A	No significant correlation between FEV1/FVC and LPC	There was no significant correlation between FEV1/FVC and LPC in BAL from patients with COPD.
Gai et al. (2022) [4]	AECOPD Patients (*n* = 58)	N/A	N/A	N/A	LPC (18:3) was significantly lower during acute exacerbations than that in recovery stage in COPD patients, especially in non-eosinophilic exacerbators.
Wang et al. (2023) [98]	AECOPD patients (*n* = 71)	N/A	N/A	N/A	LPC (16:0) and LPC (20:2) levels were increased in eosinophilic AECOPD and were associated with certain positive clinical outcomes

N/A: Data not reported in the study. LPC, Lysophosphatidylcholine; LPA, Lysophosphatidic acid; COPD, chronic obstructive pulmonary disease; FEV1, forced expiratory volume in 1 s; FVC, forced vital capacity; FEV1%pred, the percentage predicted of FEV1; BAL, bronchoalveolar lavage.

## Data Availability

Not applicable.

## References

[1] Global Initiative for Chronic Obstructive Lung Disease (2024). Global Strategy for the Diagnosis, Management, and Prevention of Chronic Obstructive Pulmonary Disease—2024 Report. https://goldcopd.org/2024-gold-report/.

[2] Wang C., Xu J., Yang L., Xu Y., Zhang X., Bai C., Kang J., Ran P., Shen H., Wen F. (2018). Prevalence and risk factors of chronic obstructive pulmonary disease in China (the China Pulmonary Health [CPH] study): A national cross-sectional study. Lancet.

[3] Rabe K.F., Watz H. (2017). Chronic obstructive pulmonary disease. Lancet.

[4] Gai X., Guo C., Zhang L., Zhang L., Abulikemu M., Wang J., Zhou Q., Chen Y., Sun Y., Chang C. (2021). Serum Glycerophospholipid Profile in Acute Exacerbation of Chronic Obstructive Pulmonary Disease. Front. Physiol..

[5] MacNee W. (2005). Pathogenesis of chronic obstructive pulmonary disease. Proc. Am. Thorac. Soc..

[6] Fischer B.M., Pavlisko E., Voynow J.A. (2011). Pathogenic triad in COPD: Oxidative stress, protease-antiprotease imbalance, and inflammation. Int. J. Chron. Obstr. Pulm. Dis..

[7] Yoon H., Shaw J.L., Haigis M.C., Greka A. (2021). Lipid metabolism in sickness and in health: Emerging regulators of lipotoxicity. Mol. Cell.

[8] Chen H., Li Z., Dong L., Wu Y., Shen H., Chen Z. (2019). Lipid metabolism in chronic obstructive pulmonary disease. Int. J. Chron. Obstr. Pulm. Dis..

[9] Tan S.T., Ramesh T., Toh X.R., Nguyen L.N. (2020). Emerging roles of lysophospholipids in health and disease. Progress. Lipid Res..

[10] Sevastou I., Kaffe E., Mouratis M.A., Aidinis V. (2013). Lysoglycerophospholipids in chronic inflammatory disorders: The PLA(2)/LPC and ATX/LPA axes. Biochim. Biophys. Acta.

[11] Rindlisbacher B., Schmid C., Geiser T., Bovet C., Funke-Chambour M. (2018). Serum metabolic profiling identified a distinct metabolic signature in patients with idiopathic pulmonary fibrosis—A potential biomarker role for LysoPC. Respir. Res..

[12] Kano K., Aoki J., Hla T. (2022). Lysophospholipid Mediators in Health and Disease. Annu. Rev. Pathol..

[13] Ackerman S.J., Park G.Y., Christman J.W., Nyenhuis S., Berdyshev E., Natarajan V. (2016). Polyunsaturated lysophosphatidic acid as a potential asthma biomarker. Biomark. Med..

[14] Bektas M., Payne S.G., Liu H., Goparaju S., Milstien S., Spiegel S. (2005). A novel acylglycerol kinase that produces lysophosphatidic acid modulates cross talk with EGFR in prostate cancer cells. J. Cell Biol..

[15] Hosogaya S., Yatomi Y., Nakamura K., Ohkawa R., Okubo S., Yokota H., Ohta M., Yamazaki H., Koike T., Ozaki Y. (2008). Measurement of plasma lysophosphatidic acid concentration in healthy subjects: Strong correlation with lysophospholipase D activity. Ann. Clin. Biochem..

[16] Yanagida K., Valentine W.J. (2020). Druggable Lysophospholipid Signaling Pathways. Adv. Exp. Med. Biol..

[17] Shea B.S., Tager A.M. (2012). Role of the lysophospholipid mediators lysophosphatidic acid and sphingosine 1-phosphate in lung fibrosis. Proc. Am. Thorac. Soc..

[18] Chun J., Hla T., Lynch K.R., Spiegel S., Moolenaar W.H. (2010). International union of basic and clinical pharmacology. LXXVIII. Lysophospholipid receptor nomenclature. Pharmacol. Rev..

[19] Turner J.A., Fredrickson M.A., D’Antonio M., Katsnelson E., MacBeth M., Van Gulick R., Chimed T.S., McCarter M., D’Alessandro A., Robinson W.A. (2023). Lysophosphatidic acid modulates CD8 T cell immunosurveillance and metabolism to impair anti-tumor immunity. Nat. Commun..

[20] Wang X., Li Y.F., Nanayakkara G., Shao Y., Liang B., Cole L., Yang W.Y., Li X., Cueto R., Yu J. (2016). Lysophospholipid Receptors, as Novel Conditional Danger Receptors and Homeostatic Receptors Modulate Inflammation-Novel Paradigm and Therapeutic Potential. J. Cardiovasc. Transl. Res..

[21] Anliker B., Chun J. (2004). Lysophospholipid G protein-coupled receptors. J. Biol. Chem..

[22] Meduri B., Pujar G.V., Durai Ananda Kumar T., Akshatha H.S., Sethu A.K., Singh M., Kanagarla A., Mathew B. (2021). Lysophosphatidic acid (LPA) receptor modulators: Structural features and recent development. Eur. J. Med. Chem..

[23] Zhao Y., Natarajan V. (2013). Lysophosphatidic acid (LPA) and its receptors: Role in airway inflammation and remodeling. Biochim. Biophys. Acta.

[24] Sheng X., Yung Y.C., Chen A., Chun J. (2015). Lysophosphatidic acid signalling in development. Development.

[25] Cummings R., Zhao Y., Jacoby D., Spannhake E.W., Ohba M., Garcia J.G., Watkins T., He D., Saatian B., Natarajan V. (2004). Protein kinase Cdelta mediates lysophosphatidic acid-induced NF-kappaB activation and interleukin-8 secretion in human bronchial epithelial cells. J. Biol. Chem..

[26] Blaho V.A., Chun J. (2018). ‘Crystal’ Clear? Lysophospholipid Receptor Structure Insights and Controversies. Trends Pharmacol. Sci..

[27] Zhao Y., He D., Zhao J., Wang L., Leff A.R., Spannhake E.W., Georas S., Natarajan V. (2007). Lysophosphatidic acid induces interleukin-13 (IL-13) receptor alpha2 expression and inhibits IL-13 signaling in primary human bronchial epithelial cells. J. Biol. Chem..

[28] Wang L., Knudsen E., Jin Y., Gessani S., Maghazachi A.A. (2004). Lysophospholipids and chemokines activate distinct signal transduction pathways in T helper 1 and T helper 2 cells. Cell. Signal..

[29] Kotarsky K., Boketoft A., Bristulf J., Nilsson N.E., Norberg A., Hansson S., Owman C., Sillard R., Leeb-Lundberg L.M., Olde B. (2006). Lysophosphatidic acid binds to and activates GPR92, a G protein-coupled receptor highly expressed in gastrointestinal lymphocytes. J. Pharmacol. Exp. Ther..

[30] Park G.Y., Lee Y.G., Berdyshev E., Nyenhuis S., Du J., Fu P., Gorshkova I.A., Li Y., Chung S., Karpurapu M. (2013). Autotaxin production of lysophosphatidic acid mediates allergic asthmatic inflammation. Am. J. Respir. Crit. Care Med..

[31] Liu P., Zhu W., Chen C., Yan B., Zhu L., Chen X., Peng C. (2020). The mechanisms of lysophosphatidylcholine in the development of diseases. Life Sci..

[32] Radu C.G., Yang L.V., Riedinger M., Au M., Witte O.N. (2004). T cell chemotaxis to lysophosphatidylcholine through the G2A receptor. Proc. Natl. Acad. Sci. USA.

[33] Yang L.V., Radu C.G., Wang L., Riedinger M., Witte O.N. (2005). Gi-independent macrophage chemotaxis to lysophosphatidylcholine via the immunoregulatory GPCR G2A. Blood.

[34] Carneiro A.B., Iaciura B.M., Nohara L.L., Lopes C.D., Veas E.M., Mariano V.S., Bozza P.T., Lopes U.G., Atella G.C., Almeida I.C. (2013). Lysophosphatidylcholine triggers TLR2- and TLR4-mediated signaling pathways but counteracts LPS-induced NO synthesis in peritoneal macrophages by inhibiting NF-κB translocation and MAPK/ERK phosphorylation. PLoS ONE.

[35] Magalhães K., Almeida P.E., Atella G., Maya-Monteiro C.M., Castro-Faria-Neto H., Pelajo-Machado M., Lenzi H.L., Bozza M.T., Bozza P.T. (2010). Schistosomal-derived lysophosphatidylcholine are involved in eosinophil activation and recruitment through Toll-like receptor-2-dependent mechanisms. J. Infect. Dis..

[36] Song M.H., Gupta A., Kim H.O., Oh K. (2021). Lysophosphatidylcholine aggravates contact hypersensitivity by promoting neutrophil infiltration and IL17 expression. BMB Rep..

[37] Barnes P.J. (2017). Cellular and molecular mechanisms of asthma and COPD. Clin. Sci. (Lond.).

[38] Zhao Y., Natarajan V. (2009). Lysophosphatidic acid signaling in airway epithelium: Role in airway inflammation and remodeling. Cell. Signal..

[39] Saatian B., Zhao Y., He D., Georas S.N., Watkins T., Spannhake E.W., Natarajan V. (2006). Transcriptional regulation of lysophosphatidic acid-induced interleukin-8 expression and secretion by p38 MAPK and JNK in human bronchial epithelial cells. Biochem. J..

[40] Zhao Y., He D., Saatian B., Watkins T., Spannhake E.W., Pyne N.J., Natarajan V. (2006). Regulation of lysophosphatidic acid-induced epidermal growth factor receptor transactivation and interleukin-8 secretion in human bronchial epithelial cells by protein kinase Cdelta, Lyn kinase, and matrix metalloproteinases. J. Biol. Chem..

[41] Zhao Y., Usatyuk P.V., Cummings R., Saatian B., He D., Watkins T., Morris A., Spannhake E.W., Brindley D.N., Natarajan V. (2005). Lipid phosphate phosphatase-1 regulates lysophosphatidic acid-induced calcium release, NF-kappaB activation and interleukin-8 secretion in human bronchial epithelial cells. Biochem. J..

[42] Rahaman M., Costello R.W., Belmonte K.E., Gendy S.S., Walsh M.T. (2006). Neutrophil sphingosine 1-phosphate and lysophosphatidic acid receptors in pneumonia. Am. J. Respir. Cell Mol. Biol..

[43] He D., Su Y., Usatyuk P.V., Spannhake E.W., Kogut P., Solway J., Natarajan V., Zhao Y. (2009). Lysophosphatidic acid enhances pulmonary epithelial barrier integrity and protects endotoxin-induced epithelial barrier disruption and lung injury. J. Biol. Chem..

[44] Takeda Y., Matoba K., Kawanami D., Nagai Y., Akamine T., Ishizawa S., Kanazawa Y., Yokota T., Utsunomiya K. (2019). ROCK2 Regulates Monocyte Migration and Cell to Cell Adhesion in Vascular Endothelial Cells. Int. J. Mol. Sci..

[45] Ray R., Rai V. (2017). Lysophosphatidic acid converts monocytes into macrophages in both mice and humans. Blood.

[46] Li Q., Wong W., Birnberg A., Chakrabarti A., Yang X., Choy D.F., Olsson J., Verschueren E., Neighbors M., Sandoval W. (2021). Lysophosphatidic acid species are associated with exacerbation in chronic obstructive pulmonary disease. BMC Pulm. Med..

[47] Gustin C., Van Steenbrugge M., Raes M. (2008). LPA modulates monocyte migration directly and via LPA-stimulated endothelial cells. Am. J. Physiol. Cell Physiol..

[48] Lundequist A., Boyce J.A. (2011). LPA5 is abundantly expressed by human mast cells and important for lysophosphatidic acid induced MIP-1β release. PLoS ONE.

[49] Yang B., Zhou Z., Li X., Niu J. (2016). The effect of lysophosphatidic acid on Toll-like receptor 4 expression and the nuclear factor-κB signaling pathway in THP-1 cells. Mol. Cell Biochem..

[50] He D., Natarajan V., Stern R., Gorshkova I.A., Solway J., Spannhake E.W., Zhao Y. (2008). Lysophosphatidic acid-induced transactivation of epidermal growth factor receptor regulates cyclo-oxygenase-2 expression and prostaglandin E(2) release via C/EBPbeta in human bronchial epithelial cells. Biochem. J..

[51] Vancheri C., Mastruzzo C., Sortino M.A., Crimi N. (2004). The lung as a privileged site for the beneficial actions of PGE2. Trends Immunol..

[52] Nakata J., Kondo M., Tamaoki J., Takemiya T., Nohara M., Yamagata K., Nagai A. (2005). Augmentation of allergic inflammation in the airways of cyclooxygenase-2-deficient mice. Respirology.

[53] Murugesan G., Sandhya Rani M.R., Gerber C.E., Mukhopadhyay C., Ransohoff R.M., Chisolm G.M., Kottke-Marchant K. (2003). Lysophosphatidylcholine regulates human microvascular endothelial cell expression of chemokines. J. Mol. Cell. Cardiol..

[54] Hara Y., Kusumi Y., Mitsumata M., Li X.K., Fujino M. (2008). Lysophosphatidylcholine upregulates LOX-1, chemokine receptors, and activation-related transcription factors in human T-cell line Jurkat. J. Thromb. Thrombolysis.

[55] Rolin J., Vego H., Maghazachi A.A. (2014). Oxidized lipids and lysophosphatidylcholine induce the chemotaxis, up-regulate the expression of CCR9 and CXCR4 and abrogate the release of IL-6 in human monocytes. Toxins.

[56] Tan M., Hao F., Xu X., Chisolm G.M., Cui M.Z. (2009). Lysophosphatidylcholine activates a novel PKD2-mediated signaling pathway that controls monocyte migration. Arterioscler. Thromb. Vasc. Biol..

[57] Schilling T., Eder C. (2009). Lysophosphatidylcholine- and MCP-1-induced chemotaxis of monocytes requires potassium channel activity. Pflug. Arch..

[58] Oestvang J., Anthonsen M.W., Johansen B. (2011). LysoPC and PAF Trigger Arachidonic Acid Release by Divergent Signaling Mechanisms in Monocytes. J. Lipids.

[59] Bach G., Perrin-Cocon L., Gerossier E., Guironnet-Paquet A., Lotteau V., Inchauspé G., Fournillier A. (2010). Single lysophosphatidylcholine components exhibit adjuvant activities in vitro and in vivo. Clin. Vaccine Immunol..

[60] Law S.H., Chan M.L., Marathe G.K., Parveen F., Chen C.H., Ke L.Y. (2019). An Updated Review of Lysophosphatidylcholine Metabolism in Human Diseases. Int. J. Mol. Sci..

[61] Nan W., Xiong F., Zheng H., Li C., Lou C., Lei X., Wu H., Gao H., Li Y. (2022). Myristoyl lysophosphatidylcholine is a biomarker and potential therapeutic target for community-acquired pneumonia. Redox Biol..

[62] Milara J., Peiró T., Serrano A., Cortijo J. (2013). Epithelial to mesenchymal transition is increased in patients with COPD and induced by cigarette smoke. Thorax.

[63] Fei J., Fu L., Cao W., Hu B., Zhao H., Li J.B. (2019). Low Vitamin D Status Is Associated with Epithelial-Mesenchymal Transition in Patients with Chronic Obstructive Pulmonary Disease. J. Immunol..

[64] Nowak-Machen M., Lange M., Exley M., Wu S., Usheva A., Robson S.C. (2015). Lysophosphatidic acid generation by pulmonary NKT cell ENPP-2/autotaxin exacerbates hyperoxic lung injury. Purinergic Signal.

[65] Ediger T.L., Schulte N.A., Murphy T.J., Toews M.L. (2003). Transcription factor activation and mitogenic synergism in airway smooth muscle cells. Eur. Respir. J..

[66] Ediger T.L., Toews M.L. (2000). Synergistic stimulation of airway smooth muscle cell mitogenesis. J. Pharmacol. Exp. Ther..

[67] Hirshman C.A., Emala C.W. (1999). Actin reorganization in airway smooth muscle cells involves Gq and Gi-2 activation of Rho. Am. J. Physiol..

[68] Toews M.L., Ediger T.L., Romberger D.J., Rennard S.I. (2002). Lysophosphatidic acid in airway function and disease. Biochim. Biophys. Acta.

[69] Zhao J., He D., Berdyshev E., Zhong M., Salgia R., Morris A.J., Smyth S.S., Natarajan V., Zhao Y. (2011). Autotaxin induces lung epithelial cell migration through lysoPLD activity-dependent and -independent pathways. Biochem. J..

[70] Decato B.E., Leeming D.J., Sand J.M.B., Fischer A., Du S., Palmer S.M., Karsdal M., Luo Y., Minnich A. (2022). LPA(1) antagonist BMS-986020 changes collagen dynamics and exerts antifibrotic effects in vitro and in patients with idiopathic pulmonary fibrosis. Respir. Res..

[71] Tager A.M., LaCamera P., Shea B.S., Campanella G.S., Selman M., Zhao Z., Polosukhin V., Wain J., Karimi-Shah B.A., Kim N.D. (2008). The lysophosphatidic acid receptor LPA1 links pulmonary fibrosis to lung injury by mediating fibroblast recruitment and vascular leak. Nat. Med..

[72] Oikonomou N., Mouratis M.A., Tzouvelekis A., Kaffe E., Valavanis C., Vilaras G., Karameris A., Prestwich G.D., Bouros D., Aidinis V. (2012). Pulmonary autotaxin expression contributes to the pathogenesis of pulmonary fibrosis. Am. J. Respir. Cell Mol. Biol..

[73] Hodge S., Hodge G., Holmes M., Reynolds P.N. (2005). Increased airway epithelial and T-cell apoptosis in COPD remains despite smoking cessation. Eur. Respir. J..

[74] Tuder R.M., Petrache I., Elias J.A., Voelkel N.F., Henson P.M. (2003). Apoptosis and emphysema: The missing link. Am. J. Respir. Cell Mol. Biol..

[75] Moolenaar W.H., Kruijer W., Tilly B.C., Verlaan I., Bierman A.J., de Laat S.W. (1986). Growth factor-like action of phosphatidic acid. Nature.

[76] Ediger T.L., Toews M.L. (2001). Dual effects of lysophosphatidic acid on human airway smooth muscle cell proliferation and survival. Biochim. Biophys. Acta.

[77] Huang L.S., Fu P., Patel P., Harijith A., Sun T., Zhao Y., Garcia J.G., Chun J., Natarajan V. (2013). Lysophosphatidic acid receptor-2 deficiency confers protection against bleomycin-induced lung injury and fibrosis in mice. Am. J. Respir. Cell Mol. Biol..

[78] Funke M., Zhao Z., Xu Y., Chun J., Tager A.M. (2012). The lysophosphatidic acid receptor LPA1 promotes epithelial cell apoptosis after lung injury. Am. J. Respir. Cell Mol. Biol..

[79] Hodge S., Hodge G., Scicchitano R., Reynolds P.N., Holmes M. (2003). Alveolar macrophages from subjects with chronic obstructive pulmonary disease are deficient in their ability to phagocytose apoptotic airway epithelial cells. Immunol. Cell Biol..

[80] Doran A.C., Yurdagul A., Tabas I. (2020). Efferocytosis in health and disease. Nat. Rev. Immunol..

[81] Barnawi J., Tran H.B., Roscioli E., Hodge G., Jersmann H., Haberberger R., Hodge S. (2016). Pro-phagocytic Effects of Thymoquinone on Cigarette Smoke-exposed Macrophages Occur by Modulation of the Sphingosine-1-phosphate Signalling System. COPD.

[82] Morimoto K., Janssen W.J., Fessler M.B., McPhillips K.A., Borges V.M., Bowler R.P., Xiao Y.Q., Kench J.A., Henson P.M., Vandivier R.W. (2006). Lovastatin enhances clearance of apoptotic cells (efferocytosis) with implications for chronic obstructive pulmonary disease. J. Immunol..

[83] Kim E.A., Kim J.A., Park M.H., Jung S.C., Suh S.H., Pang M.G., Kim Y.J. (2009). Lysophosphatidylcholine induces endothelial cell injury by nitric oxide production through oxidative stress. J. Matern. Fetal Neonatal Med..

[84] Wang Y., Wang Y., Li G.R. (2016). TRPC1/TRPC3 channels mediate lysophosphatidylcholine-induced apoptosis in cultured human coronary artery smooth muscles cells. Oncotarget.

[85] Yang S., Chen J., Ma B., Wang J., Chen J. (2022). Role of Autophagy in Lysophosphatidylcholine-Induced Apoptosis of Mouse Ovarian Granulosa Cells. Int. J. Mol. Sci..

[86] Tanosaki T., Mikami Y., Shindou H., Suzuki T., Hashidate-Yoshida T., Hosoki K., Kagawa S., Miyata J., Kabata H., Masaki K. (2022). Lysophosphatidylcholine Acyltransferase 1 Deficiency Promotes Pulmonary Emphysema via Apoptosis of Alveolar Epithelial Cells. Inflammation.

[87] Lauber K., Bohn E., Krober S.M., Xiao Y.J., Blumenthal S.G., Lindemann R.K., Marini P., Wiedig C., Zobywalski A., Baksh S. (2003). Apoptotic cells induce migration of phagocytes via caspase-3-mediated release of a lipid attraction signal. Cell.

[88] Dan Dunn J., Alvarez L.A., Zhang X., Soldati T. (2015). Reactive oxygen species and mitochondria: A nexus of cellular homeostasis. Redox Biol..

[89] Shao Y., Nanayakkara G., Cheng J., Cueto R., Yang W.Y., Park J.Y., Wang H., Yang X. (2018). Lysophospholipids and Their Receptors Serve as Conditional DAMPs and DAMP Receptors in Tissue Oxidative and Inflammatory Injury. Antioxid. Redox Signal.

[90] Inoue N., Takeshita S., Gao D., Ishida T., Kawashima S., Akita H., Tawa R., Sakurai H., Yokoyama M. (2001). Lysophosphatidylcholine increases the secretion of matrix metalloproteinase 2 through the activation of NADH/NADPH oxidase in cultured aortic endothelial cells. Atherosclerosis.

[91] Ares M.P., Kallin B., Eriksson P., Nilsson J. (1995). Oxidized LDL induces transcription factor activator protein-1 but inhibits activation of nuclear factor-kappa B in human vascular smooth muscle cells. Arterioscler. Thromb. Vasc. Biol..

[92] Ojala P.J., Hirvonen T.E., Hermansson M., Somerharju P., Parkkinen J. (2007). Acyl chain-dependent effect of lysophosphatidylcholine on human neutrophils. J. Leukoc. Biol..

[93] Vázquez-Medina J.P., Dodia C., Weng L., Mesaros C., Blair I.A., Feinstein S.I., Chatterjee S., Fisher A.B. (2016). The phospholipase A2 activity of peroxiredoxin 6 modulates NADPH oxidase 2 activation via lysophosphatidic acid receptor signaling in the pulmonary endothelium and alveolar macrophages. FASEB J..

[94] Naz S., Kolmert J., Yang M., Reinke S.N., Kamleh M.A., Snowden S., Heyder T., Levänen B., Erle D.J., Sköld C.M. (2017). Metabolomics analysis identifies sex-associated metabotypes of oxidative stress and the autotaxin-lysoPA axis in COPD. Eur. Respir. J..

[95] Li Q., Wong W.R., Chakrabarti A., Birnberg A., Yang X., Verschueren E., Neighbors M., Rosenberger C., Grimbaldeston M., Tew G.W. (2021). Serum Lysophosphatidic Acid Measurement by Liquid Chromatography-Mass Spectrometry in COPD Patients. J. Am. Soc. Mass Spectrom..

[96] Halper-Stromberg E., Gillenwater L., Cruickshank-Quinn C., O’Neal W.K., Reisdorph N., Petrache I., Zhuang Y., Labaki W.W., Curtis J.L., Wells J. (2019). Bronchoalveolar Lavage Fluid from COPD Patients Reveals More Compounds Associated with Disease than Matched Plasma. Metabolites.

[97] Cruickshank-Quinn C.I., Jacobson S., Hughes G., Powell R.L., Petrache I., Kechris K., Bowler R., Reisdorph N. (2018). Metabolomics and transcriptomics pathway approach reveals outcome-specific perturbations in COPD. Sci. Rep..

[98] Wang Y., Chang C., Tian S., Wang J., Gai X., Zhou Q., Chen Y., Gao X., Sun Y., Liang Y. (2023). Differences in the lipid metabolism profile and clinical characteristics between eosinophilic and non-eosinophilic acute exacerbation of chronic obstructive pulmonary disease. Front. Mol. Biosci..

[99] Blom M., Tool A.T., Wever P.C., Wolbink G.J., Brouwer M.C., Calafat J., Egesten A., Knol E.F., Hack C.E., Roos D. (1998). Human eosinophils express, relative to other circulating leukocytes, large amounts of secretory 14-kD phospholipase A2. Blood.

[100] Nishiyama O., Kume H., Kondo M., Ito Y., Ito M., Yamaki K. (2004). Role of lysophosphatidylcholine in eosinophil infiltration and resistance in airways. Clin. Exp. Pharmacol. Physiol..

[101] Zhu X., Learoyd J., Butt S., Zhu L., Usatyuk P.V., Natarajan V., Munoz N.M., Leff A.R. (2007). Regulation of eosinophil adhesion by lysophosphatidylcholine via a non-store-operated Ca^2+^ channel. Am. J. Respir. Cell Mol. Biol..

[102] Gan L., Xue J.X., Li X., Liu D.S., Ge Y., Ni P.Y., Deng L., Lu Y., Jiang W. (2011). Blockade of lysophosphatidic acid receptors LPAR1/3 ameliorates lung fibrosis induced by irradiation. Biochem. Biophys. Res. Commun..

